# Recent Advances in the Electrochemical Biosensing of DNA Methylation

**DOI:** 10.3390/ijms26136505

**Published:** 2025-07-06

**Authors:** Sanu K. Anand, Robert Ziółkowski

**Affiliations:** Medical Biotechnology, Faculty of Chemistry, Warsaw University of Technology, Stanisława Noakowskiego 3, 00-664 Warsaw, Poland; sanukanand@gmail.com

**Keywords:** DNA methylation, epigenetic modification, electrochemical sensor, electrooxidation, restriction endonuclease, 5-methylcytosine antibody

## Abstract

DNA methylation, as a critical epigenetic modification, plays a central role in gene regulation and has emerged as a powerful biomarker for early disease diagnostics, particularly in cancer. Owing to the limitations of traditional bisulfite sequencing—such as high cost, complexity, and chemical degradation—electrochemical biosensors have gained substantial attention as promising alternatives. This review summarizes recent advancements in electrochemical platforms for bisulfite-free detection of DNA methylation, encompassing direct oxidation strategies, enzyme-assisted recognition (e.g., restriction endonucleases and methyltransferases), immunoaffinity-based methods, and a variety of signal amplification techniques such as rolling circle amplification and catalytic hairpin assembly. Additional approaches, including strand displacement, magnetic enrichment, and adsorption-based detection, are also discussed. These systems demonstrate exceptional sensitivity, often down to the attomolar or femtomolar level, as well as high selectivity, reproducibility, and suitability for real biological matrices. The integration of nanomaterials and redox-active probes further enhances analytical performance. Importantly, many of these biosensing platforms have been validated using clinical samples, reinforcing their translational relevance. The review concludes by outlining current challenges and future directions, emphasizing the potential of electrochemical biosensors as scalable, cost-effective, and minimally invasive tools for real-time epigenetic monitoring and early-stage disease diagnostics.

## 1. Introduction

Epigenetics, derived from the term “above genetics,” refers to heritable modifications in gene expression that occur without changes to the underlying DNA sequence [1,2]. These modifications, collectively termed epigenetic alterations, typically encompass DNA methylation, post-translational modifications of histone proteins, and regulatory influences exerted by non-coding RNAs [3,4,5]. Among these, DNA methylation represents the most extensively studied epigenetic marker. It involves the enzymatic transfer of a methyl group from S-adenosylmethionine to either adenine (A) or cytosine (C) residues [6]. In eukaryotes, this covalent modification predominantly targets the C5 position of cytosine within CpG dinucleotides, resulting in the formation of 5-methylcytosine (5-mC), a process catalyzed by DNA methyltransferases [7,8,9,10]. DNA methylation plays a pivotal role in the regulation of genetic and cellular processes and is increasingly recognized as a key biomarker in the early detection of various diseases [5]. Numerous studies have demonstrated that hypermethylation can lead to aberrant silencing of tumor suppressor genes, thereby contributing to oncogenesis [11]. Elevated levels of hypermethylated circulating free DNA (cfDNA) are detectable in the bloodstream of cancer patients compared to healthy individuals, exhibiting preferential adsorption to specific substrates—a property that facilitates its use in diagnostic screening [12]. Notably, such aberrant methylation often arises during the initial stages of tumor development, reinforcing its potential as a clinically valuable tool for early cancer diagnostics [13].

As with other biomarkers, DNA methylation can be assessed in various biological matrices, including body fluids—where it exists in nanogram-per-milliliter concentrations under pathological conditions—as well as in frozen, formalin-fixed, or paraffin-embedded tissue specimens. This versatility underscores the growing preference for DNA-based “epimarks” over conventional protein biomarkers in disease diagnostics [1]. Given these attributes, the continuous and accurate monitoring of DNA methylation is of special importance in pathological research.

Bisulfite sequencing is regarded as the gold standard for detecting DNA methylation. This method differentiates between methylated and unmethylated cytosines by converting the latter into uracil, while methylated cytosines remain unaltered [14]. Although initially recognized for its accuracy in mapping methylation patterns, this technique suffers from several limitations, including incomplete conversion, limited sensitivity, high cost, and the need for sophisticated instrumentation, which hinder its routine clinical application [15]. In recent years, significant efforts have been made to develop alternative sequencing methodologies capable of overcoming these challenges.

One of the emerging and promising areas of research involves the advancement of biosensor technologies, which offer a combination of simplicity and reliability while maintaining the analytical accuracy of conventional methodologies. In such systems, the specific interaction between a receptor and its target analyte is converted into a quantifiable signal by means of various transduction mechanisms, including optical and electrochemical platforms. Biosensors effectively address several limitations associated with traditional analytical approaches, such as high-performance liquid chromatography (HPLC) and capillary electrophoresis, which often involve complex protocols, require large sample volumes, and rely on costly instrumentation. Optical biosensors, in particular, stand out as sensitive analytical tools capable of detecting changes in light absorption, reflection, or emission, which are directly proportional to the concentration of the analyte of interest [10]. This enables precise quantitative analysis. Depending on the nature of the transduction mechanism, optical assays may employ fluorescence (detecting changes in emission intensity or wavelength upon target interaction), colorimetry (observing color changes in the presence of an analyte), surface plasmon resonance (measuring refractive index shifts), or surface-enhanced Raman scattering (SERS), which detects alterations in vibrational properties following analyte adsorption on a nanostructured substrate [10,16]. Among these techniques, fluorescence-based sensing exhibits particularly high intrinsic sensitivity, rendering it a highly attractive strategy within the broader spectrum of optical biosensing modalities. Numerous optical biosensing methodologies with superior analytical performance have been developed. For example, Yu et al. reported a fluorescence-based approach for site-specific DNA methylation detection with a femtomolar (fM) detection limit [17]. Similarly, Cao et al. used enhanced fluorescence sensitivity using redox-cycling signal amplification, achieving fM-level detection of methylated DNA [18].

Despite these advancements, the integration of such highly sensitive methods into portable and miniaturized platforms remains a significant challenge, thereby restricting their broader application—particularly in the field of clinical diagnostics.

As an emerging alternative, electrochemical biosensors have attracted considerable interest due to their distinctive advantages. These platforms are recognized for their simplicity, rapid response time, low cost, portability, and compatibility with miniaturized formats—qualities that are particularly desirable for point-of-care diagnostics and high-throughput clinical screening [19]. Moreover, electrochemical transduction offers high specificity and sensitivity, making it exceptionally suitable for detecting minute concentrations of methylated DNA in complex biological matrices. Unlike traditional techniques, these systems operate without extensive sample pretreatment, rely on straightforward instrumentation, and are amenable to multiplexing and integration into lab-on-chip devices [11,20,21,22].

Recent advances have led to the development of bisulfite-free electrochemical biosensing platforms for methylation analysis. These include both label-free and label-based strategies [23]. One such approach exploits the electrochemical differences between cytosine and 5-mC, enabling direct detection without chemical pretreatment [24]. Alternatively, ultrasensitive detection has been achieved through the use of enzymatic or antibody-based labels [24]. Restriction enzyme-based methods employ methylation-sensitive or -resistant endonucleases to cleave DNA at specific sites, with subsequent analysis of electrochemical signals from cleavage products [25]. Immunoaffinity assays utilize antibodies specific to 5-mC to enrich methylated sequences for detection [26].

Given the low abundance of methylated DNA in clinical samples, signal amplification strategies have been integrated to enhance detection sensitivity [27]. Additionally, several innovative biosensing architectures that fall outside traditional classifications have demonstrated excellent analytical performance.

Sensitivity remains a critical parameter in the development of effective DNA-based biosensors. Gold electrodes are commonly employed due to the strong affinity between gold surfaces and thiol- or amine-labeled DNA probes [25,28]. Beyond unmodified electrodes, a variety of surface modifications have been implemented to reduce overpotentials, increase signal intensity, enhance analyte capture, and minimize interference from extraneous species [29,30,31]. Among these, gold nanoparticles (AuNPs) and their composites are particularly prevalent, as they readily bind to thiol-functionalized DNA [32,33]. In addition, numerous nanostructured materials—such as magnetic nanoparticles, conductive polymers, and composite materials—have shown significant promise in this domain [34,35]. For instance, polyvinyl alcohol combined with reduced graphene oxide (rGO) has been used to efficiently immobilize anti-5-mC antibodies [36]. Similarly, polypyrrole doped with multi-walled carbon nanotubes (MWCNTs), and a composite of graphene oxide (GO), Fe_3_O_4_, and β-cyclodextrin, have demonstrated high sensitivity in methylation analysis [37]. These novel materials act as effective signal amplifiers, enabling the ultrasensitive detection of low-abundance targets such as methylated DNA [38].

Epigenomic modifications have attracted substantial scientific attention, as reflected by the growing number of publications in recent years. This review highlights recent advances in bisulfite-free electrochemical biosensing technologies for the detection of epigenetic alterations, offering an up-to-date overview of innovative platforms developed for this purpose and emphasizing their superior analytical characteristics and translational potential.

## 2. Electrochemical Sensing of DNA Methylation

Conventional DNA sequencing techniques are inherently limited in their ability to detect DNA methylation, as both cytosine (C) and its methylated derivative, 5-methylcytosine (5-mC), retain identical Watson–Crick base-pairing properties [39]. Consequently, a variety of advanced sensing strategies have been developed, which are detailed in the following sections.

### 2.1. Direct Electrochemical Oxidation of 5-Methylcytosine

Since individual nucleobases possess distinct oxidation potentials at specific electrode surfaces, electrochemical detection based on their redox behavior offers a promising approach for identifying 5-mC [24]. However, the similar molecular structures of C and 5-mC, and the close proximity of their oxidation potentials—especially relative to thymine—often result in significant signal overlap and analytical interference [21]. To address these challenges, researchers have developed innovative electrode materials with extended potential windows and enhanced electrical conductivity.

**Figure 1:**
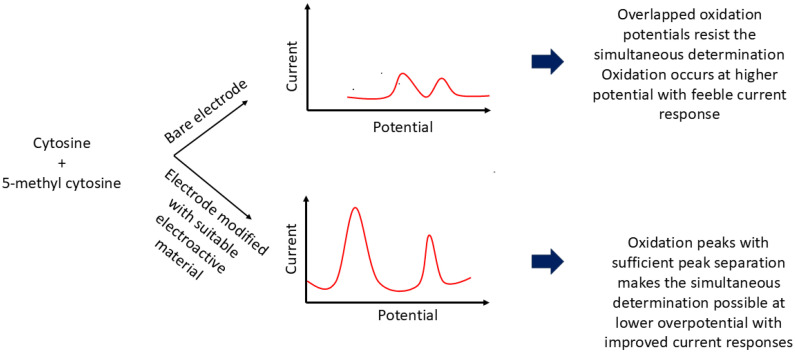
Schematic illustration of the electrochemical responses of cytosine and 5-mC at bare versus modified electrodes.

In 2010, Niwa et al. introduced a nanocarbon film electrode fabricated via electron cyclotron resonance sputtering, which successfully discriminated between C and 5-mC with a peak separation of 160 mV [39]. This platform enabled quantification of methylation in the *retinoblastoma* gene, a well-known ocular tumor marker, independent of methylation site position. Similarly, Zou and colleagues (2010) reported a rapid, label-free method based on direct electrocatalytic oxidation of nucleobases using a glassy carbon electrode (GCE) modified with a choline chloride monolayer and multi-walled carbon nanotubes (MWCNTs) [9]. The oxidation signals of 5-mC and C were resolved into two distinct peaks with a separation of 170 mV. The methyl group’s electron-donating character facilitated oxidation at the C5–C6 double bond, reducing the overpotential to 1.16 V (versus 1.33 V for C). However, this method could not adequately separate the overlapping signals of 5-mC and thymine. To address this, the authors accounted for adenine’s contribution—present in equimolar amounts to thymine—thereby isolating the signal of 5-mC and achieving a detection range of 0.60 to 450 µM, with a limit of detection (LOD) of 0.15 µM. The same research group later demonstrated the ability to discriminate among adenine, guanine, thymine, cytosine, and 5-mC using a polypyrrole–MWCNT composite film on a modified GCE [40]. This nanocomposite featured a highly oriented structure with abundant edge-plane-like active sites, which enhanced electron transfer kinetics and voltammetric responses. Notably, the method resolved C and 5-mC with a peak separation of 180 mV and was successfully applied to salmon sperm DNA, where oxidation peaks for G, A, and C were well-defined. While 5-mC and T remained overlapped, their quantification was achieved indirectly through the known stoichiometric relationship between G and the combined 5-mC/C content. Figure 1 schematically represents the electrochemical behavior of C and 5-mC at both bare and modified electrodes.

In 2015, Travas-Sejdic et al. introduced a simplified approach based on electrochemical impedance spectroscopy (EIS) to differentiate methylated and unmethylated DNA [41]. An amino-functionalized DNA probe was immobilized on an electroactive copolymer substrate. Denatured double-stranded DNA (dsDNA) was used as the target, and discrimination was achieved by comparing charge transfer resistance (R_ct_) values. Methylated DNA, due to its reduced hybridization efficiency, exhibited lower R_ct_ values than its unmethylated counterparts. Moreover, probe density was found to influence hybridization: higher densities increased steric hindrance for methylated DNA, leading to diminished impedimetric response. This study laid the groundwork for further sensor design innovations for methylation analysis.

Given the growing interest in 5-hydroxymethylcytosine (5-hmC) as a clinically relevant epigenetic biomarker, several electrochemical strategies have emerged to selectively detect this base. Topal et al. recently developed a simple and effective method using a pencil graphite electrode under optimized anodic stripping conditions [42]. 5-hmC was detected at 1.26 V in a linear range of 4 × 10^−6^ to 2 × 10^−4^ M, while C was oxidized at 1.33 V, facilitating clear differentiation. They also demonstrated simultaneous quantification of 5-mC in the presence of 4.0 × 10^−5^ M C in human urine, supporting the sensor’s applicability in real biological samples. In another example, Lim et al. employed the two-dimensional material MXene for sensitive detection of 5-hmC [43]. A flexible sensor was fabricated by printing silver ink on polyethylene terephthalate (PET) via nozzle-jet printing, followed by deposition of a composite comprising gold nanoparticles and MXene. The negatively charged –OH groups on MXene hindered target adsorption, thereby enhancing current response and enabling detection at picomolar levels. Importantly, the method demonstrated a 1.8-fold higher current response for 5-hmC compared to 5-mC, illustrating its selectivity toward these structurally similar epigenetic variants.

Despite these advances, the major challenge remains ensuring high specificity in the presence of structurally analogous nucleobases, especially in complex biological samples. This issue can be mitigated through the incorporation of selective recognition elements—such as molecular labels, antibodies, or aptamers—into the receptor interface, which continues to drive further innovation in this field.

### 2.2. Restriction Endonuclease-Based Assay

**Figure 2:**
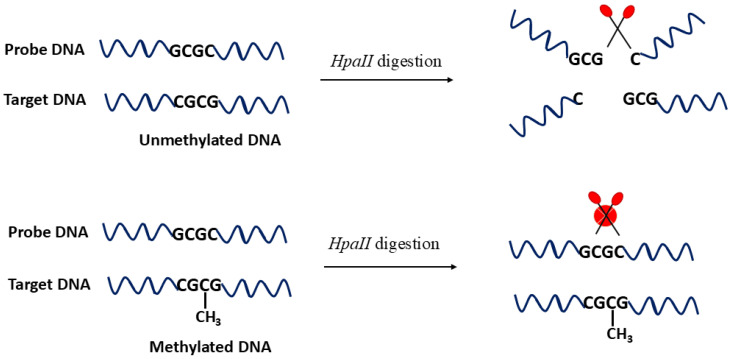
Schematic representation of the specific *HpaII* digestion in a DNA duplex. *HpaII* cleaves the C–G pair in unmethylated DNA (upper part of the figure), and such cleavage is blocked when the internal C is methylated (lower part of the figure).

The use of restriction endonucleases for the detection of DNA methylation has constituted a significant milestone in epigenetic research. These enzymes have been extensively adopted by the scientific community to identify methylated DNA with high sequence specificity. Restriction endonucleases are broadly classified into two categories: methylation-sensitive and methylation-dependent (or -resistant) enzymes [1]. For instance, enzymes such as *BstUI, HpaII*, and *DpnI* belong to the methylation-sensitive class and selectively cleave specific base sequences in unmethylated DNA, whereas their activity is blocked in the presence of methylated cytosines [44,45]. Among these, *HpaII* is particularly well studied, and its enzymatic action is schematically illustrated in Figure 2, where *HpaII* cleaves the C–G pair in unmethylated DNA (upper part of the figure) and such cleavage is blocked when the internal C is methylated (lower part of the figure). This detection specificity allows *HpaII* to be used as an efficient probe to analyze methylation at the CpG sites. In contrast, *GlaI* is a methylation-dependent enzyme that exclusively recognizes methylated sequences, leaving unmethylated DNA intact [27,46]. Although such enzymes offer excellent sequence specificity, their application is inherently restricted to the presence of well-defined recognition sites. Therefore, successful implementation often requires multiple recognition sequences to ensure analytical reliability and accuracy [27]. To increase coverage and resolution, the simultaneous use of multiple endonucleases has been proposed, resulting in complete site-specific digestion and thereby enabling highly sensitive and accurate analysis [47].

**Figure 3:**
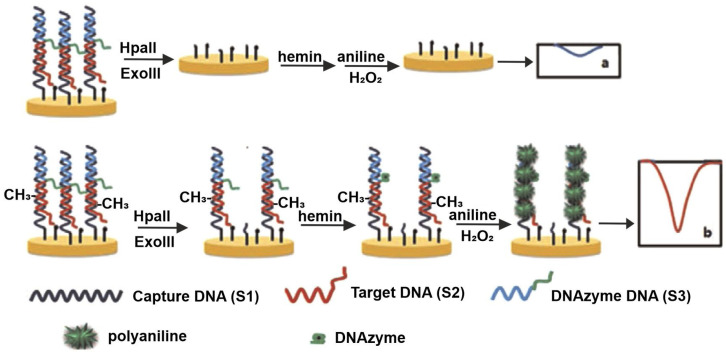
Mechanism of HpaII-based sensing using DNAzyme-assisted electropolymerization. Upper part of the figure shows situation with unmethylated DNA where combination of nucleases (*HpaII*, *Exo III*) digests the receptor layer, resulting in small redox peak during electrochemical analysis. Lower part of the figure shows opposite situation, where the nucleases’ activity is blocked by methylated DNA. Reprinted with permission from (Wei et al., 2015) [49].

Gooding and coworkers demonstrated a pioneering approach that coupled the sequence specificity of *HhaI endonuclease* with the high surface reactivity of dispersible electrodes to achieve the ultrasensitive detection of methylated DNA in human blood samples [44]. They employed gold-coated magnetic nanoparticles as conductive carriers for the immobilization of thiolated DNA probes. After hybridization with the target, the DNA duplex was magnetically separated and exposed to the restriction enzyme. Using [Fe(CN)_6_]^3−^ as an electrochemical probe, the successive assay steps were monitored. For unmethylated DNA, the *HhaI*-mediated cleavage reduced steric hindrance and surface charge repulsion, resulting in an increased peak current, while methylated DNA remained intact, exhibiting a suppressed signal. This sensor achieved an impressive detection range from 2 aM to 20 nM and was successfully validated in clinical samples. In another study, Richtera et al. compared the performance of various nanocomposites—reduced graphene oxide (RGO) combined with Ag, Au, or Cu nanoparticles—for the selective detection of methylation patterns [48]. Single-stranded DNA (ssDNA) was immobilized onto the electrode via metal–sulfur interactions, followed by hybridization and in vitro methylation using *M.SssI methyltransferase*. Upon treatment with *HpaII*, only unmethylated duplexes were cleaved. Among the tested composites, RGO–AuNPs exhibited the most sensitive response due to the strong thiol–gold affinity, achieving a detection range of 5–500 U mL^−1^ and a limit of detection (LOD) of 0.04 U mL^−1^**.** The platform demonstrated excellent compatibility with human serum, underscoring its clinical potential. A novel strategy was introduced by Wei et al., employing polyaniline (PANI) deposition guided by a horseradish peroxidase (HRP)-mimicking DNAzyme for the detection of both methylation and methyltransferase (MTase) activity [49]. In this method, captured DNA was immobilized and hybridized with both target DNA and a G-rich sequence. The *HpaII* action leaves some short dsDNA in the sample, and its complete digestion was ensured by the combined action of *HpaII* and *Exonuclease III (Exo III)*; hence, the G-rich DNA—responsible for catalyzing PANI polymerization—was removed, resulting in reduced current response. Conversely, in the presence of MTase, methylation inhibited *HpaII* digestion, allowing PANI deposition to proceed and generating a strong electrochemical signal. This sensor offered a linear detection range of 0.5–60 U mL^−1^ with an LOD of 0.12 U mL^−1^. The sensing behavior is schematically represented in Figure 3.

This enzyme-assisted strategy was further extended to monitor DNA demethylation and demethylase activity [50]. In this case, hemi-methylated DNA probes were immobilized on a gold electrode, and methylation states were probed via ferrocene carboxylic acid tags at the 3′ end. In the absence of demethylase, methylated sites blocked *BstUI* activity, preserving the redox label and maintaining the signal. Upon demethylation, cleavage by *BstUI* released the ferrocene tag, resulting in a reduced current. The assay displayed a linear range of 0.5 to 500 ng mL^−1^ and an LOD of 0.17 ng mL^−1^, with minimal cross-reactivity to other proteins, including MBD1, MBD4, MeCP2, HSA, and MCF-7, thereby affirming high selectivity in complex biological environments.

In a more recent development, Chen et al. [51] explored the methylation-to-demethylation pathway mediated by the TET1 enzyme, using *MspI* restriction sites as a readout [51]. TET1 catalyzes the oxidation of 5-mC to 5-hmC and further derivatives, ultimately reverting it to cytosine. In this strategy, a semi-methylated G-rich dsDNA-modified GCE was used with H_2_O_2_–hydroquinone redox reporters. *MspI* selectively cleaved the unmethylated sites, liberating G-rich sequences that formed a hemin–DNAzyme complex, catalyzing the H_2_O_2_ reaction and yielding an electrochemical signal. Upon TET1 activity, the methylated state was lost, and *MspI* cleavage was inhibited, thus blocking signal development. The system exhibited a linear response to TET1 from 0.7 to 10.5 ng µL^−1^, with an LOD of 0.027 ng µL^−1^.

While restriction enzyme-based approaches offer excellent sequence specificity and sensitivity, they are inherently limited by enzyme recognition site-dependence and sensitivity to assay conditions, which may reduce applicability to certain clinical contexts. Nevertheless, the integration of enzyme biorecognition with electrochemical detection provides a powerful hybrid platform, enabling robust signal amplification, label-free operation, and high diagnostic utility, especially when used in combination with nanomaterials or redox-active tags.

### 2.3. Immuno/Affinity-Based Assay

**Figure 4:**
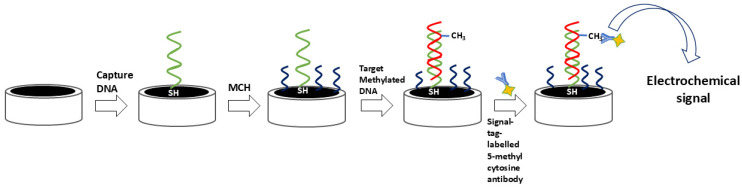
Schematic of an antibody-mediated electrochemical approach for the recognition of methylated DNA.

Immunoaffinity-based strategies constitute a bisulfite- and enzyme-free approach for DNA methylation detection, leveraging the specific recognition of 5-methylcytosine (5-mC) by anti-5-mC antibodies (anti-5-mC) [52,53]. These antibodies selectively bind to both single- and double-stranded methylated DNA, enabling a range of ultrasensitive immunoquantification protocols. The analytical performance of these systems is enhanced not only by the antibody specificity but also by the careful selection of substrate materials, immobilization strategies, and labeling methods, which together dictate signal strength and detection range [52]. Frequently, enzymes such as horseradish peroxidase (HRP) and glucose oxidase (GOx) serve as signal transducers, facilitating the electrochemical readout of antibody–target interactions [54]. Additionally, many signal tags, particularly innovative nanomaterials, are used as labels for antibodies. Such tags can amplify the signal, thereby enabling superior analytical characteristics. A simple immune approach employing a signal-tag-labeled antibody is shown in Figure 4.

**Figure 5:**
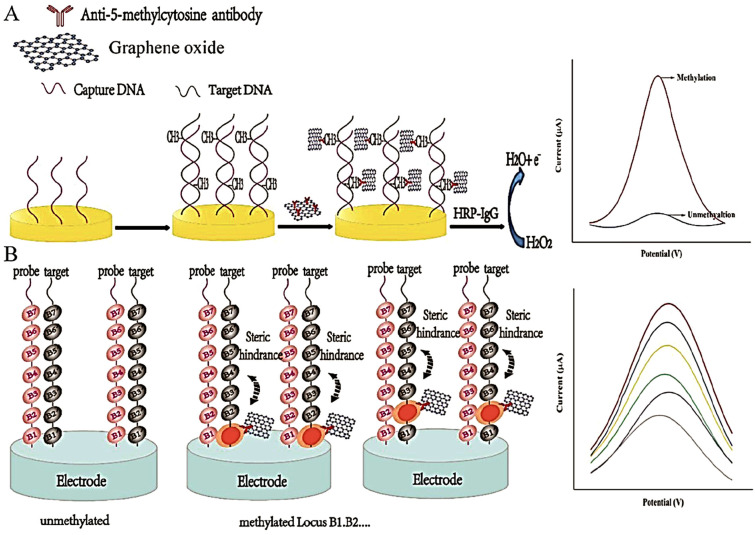
(**A**) Electrochemical immunoquantification of DNA methylation using secondary HRP-IgG. (**B**) Effect of steric hindrance on the electrochemical response after interacting with different methylated sites on the surface of electrode. Reprinted with permission from [2].

For example, Ai et al. combined the methylation-selective activity of *M.SssI* methyltransferase (MTase) with the targeting ability of anti-5-mC and the electrocatalytic properties of HRP-conjugated immunoglobulins to quantify DNA methylation [21]. Thiolated DNA was immobilized on an AuNP-modified glassy carbon electrode (GCE), hybridized with complementary sequences, and methylated in situ. The resulting duplexes were then selectively bound by anti-5-mC, followed by HRP-IgG. The signal originated from HRP-catalyzed oxidation of hydroquinone to benzoquinone. Specificity was validated using *HpaII* digestion (blocked by methylation) and mismatch discrimination. This method underscored the potential of enzyme–antibody–nanoparticle combinations for high-fidelity methylation detection. Zheng et al. expanded on this concept by anchoring anti-5-mC antibodies onto carboxyl-functionalized graphene oxide (GO) nanosheets [2]. A secondary HRP-IgG amplified the electrochemical signal, producing a detection limit of 0.84 fM over a linear range of 10^−15^ to 10^−8^ M. Variations in current response were attributed to methylation-dependent steric hindrance affecting antibody binding. The sensing protocol of this method is demonstrated as Figure 5. Similarly, Shiddiky et al. employed bioengineered polyhydroxybutyrate nanobeads functionalized with anti-5-mC-HRP conjugates to facilitate amperometric detection of methylated DNA in ovarian cancer cells [20]. Signal reduction was linked to benzoquinone–DNA interactions, which impeded redox probe diffusion and introduced electrostatic repulsion. The method successfully discriminated between cancerous (SKOV-3, OVCAR-3) and non-cancerous (MeT-5A) cell lines, highlighting its clinical point-of-care relevance.

The optoelectronic synergy of metal nanoparticles remains a cornerstone in the design of high-performance immunosensors. Their multifaceted characteristics continue to make them indispensable candidates, either alone or as a composite with any other promising materials, even in the current technological landscape. Such nanocomposites display amplified electrochemical responses via the synergistic coupling of their individual counterparts. Zhu et al. reported a hybrid platform combining AuNPs, reduced graphene oxide (RGO), and graphite carbon nitride, conjugated with anti-5-mC, to achieve femtomolar detection levels [14]. Binding efficiency was validated by varying the number of methylated sites, further confirming the robustness of nanocomposite-supported antibody immobilization. Geng et al. used AgNP-decorated carbon nanotubes to tag anti-5-mC antibodies [55]. Methylation was induced by M.SssI MTase, and quantification was performed via linear sweep voltammetry (LSV). A strong LSV signal directly correlated with MTase activity and methylation levels, offering a wide detection range (0.05–120 U/mL) with an LOD of 0.03 U/mL. The amplified and reproducible response was attributed to the electrocatalytic properties and structural integrity of the AgNP-CNT composite.

In a label-free format, Safarzadeh et al. proposed an immunosensor based on aminated RGO electrodes, where the surface amino groups served as anchors for anti-5-mC [7]. Upon DNA hybridization and methylation capture, redox probe ([Fe(CN)_6_]^3−^) current decreased due to surface blocking, then increased following immunocomplex formation, enabling femtomolar-level detection. Superior performance with double-stranded DNA (dsDNA) indicated excellent target selectivity. Teixeira et al. introduced an innovative system based on the reduction of dissolved oxygen at a fluorine-doped tin oxide (FTO) electrode, modified via electropolymerization of Bismarck Brown Y and HAuCl_4_, followed by anti-5-mC immobilization [56]. Methylated DNA binding reduced oxygen availability, resulting in increased impedance in PBS solutions. The interplay between electrocatalytic AuNPs and polymer-anchored amine groups enabled a detection limit as low as 0.64 pg/mL, confirming the system’s electroanalytical precision.

Magnetic nanomaterials also offer strategic advantages in immunoaffinity assays due to their high biomolecule-loading capacity, effective dispersion under agitation, and suitability for isolating low-abundance biomarkers [53]. In a study by Shiddiky et al., eco-friendly starch-assisted synthesis of carboxyl-functionalized Fe_3_O_4_ nanoparticles allowed covalent conjugation with amine-labeled anti-5-mC via EDC/NHS chemistry [52]. After target capture, magnetic separation, and electrode deposition, methylation levels were quantified using [Ru(NH_3_)_6_]^3+^ in DPV mode. The system discriminated DNA methylation levels down to 5%, with clear resolution between cancerous and noncancerous profiles. The authors have substantiated the selectivity of the system via the enhanced current density of completely methylated DNA target compared to its nonmethylated samples. They have addressed the main clinical challenges in DNA methylation quantification by determining as low as 5% methylation and also the different methylation levels in normal as well as cancer cell lines. In another strategy, streptavidin-coated magnetic beads were functionalized with anti-5-mC and hybridized with denatured DNA and GOx-conjugated secondary antibodies [53]. Detection was performed at Prussian blue-modified screen-printed electrodes via amperometric monitoring of H_2_O_2_ generation in glucose buffer. Selectivity was validated by comparing current responses from methylated and nonmethylated targets, with negligible signal from the latter. This affinity-enriched magnetic sensing platform provides a powerful and scalable tool for clinical diagnostics, capable of integrating high specificity with electrochemical sensitivity.

### 2.4. Other Emerging Electrochemical Methods

This section highlights novel electrochemical sensing strategies for DNA methylation that do not strictly fall within the previously discussed categories but nonetheless demonstrate excellent analytical performance and offer promising clinical applicability.

Zhao et al. proposed a simple yet effective approach to cancer screening by exploiting the preferential adsorption of methylated circulating free DNA (cfDNA) onto gold surfaces, using the Septin9 gene as a colorectal cancer biomarker [12]. In cancer patients, cfDNA exhibits higher methylation levels, leading to increased hydrophobicity and consequently stronger adsorption to the gold electrode. Using square wave voltammetry (SWV) with ferrocene as the redox indicator, a decrease in current was observed in the presence of methylated DNA. The platform demonstrated robust discrimination between 200 cancer patients and 100 healthy controls, confirming excellent stability, reproducibility, and real-world relevance.

In 2022, Zheng et al. developed a strategy based on the differential strand displacement efficiency of methylated versus unmethylated DNA [15]. Methylation disrupts base-pair stability, thus leading to increased susceptibility to strand displacement. To enhance this effect, they utilized peptide nucleic acids (PNAs), which have reduced electrostatic repulsion compared to DNA, thereby increasing displacement efficiency. After immobilizing capture probes on a gold electrode, ferrocene-labeled PNA probes were introduced to detect displacement events. This method achieved a detection limit of 0.075 pM across a range of 10^−14^ to 10^−7^ M and was successfully validated in human blood and breast cancer cell samples, demonstrating practical utility and biocompatibility.

Liang et al. introduced a unique methodology combining immunomagnetic beads and magnetically modulated electrochemical detection, utilizing Prussian blue as a redox marker [57]. Movement of beads under an external magnetic field created ON–OFF switching states at the electrode surface, producing periodic current signals. Applying Fast Fourier Transform (FFT) enabled the extraction of weak frequency-domain signals otherwise buried in background noise. This system achieved detection as low as 0.5 pg of methylated DNA and was successfully applied to hepatocellular carcinoma cell samples, proving its effectiveness in low-volume clinical diagnostics.

Many studies have evolved based on the electrostatic interactions between the polycationic center, like polyethylenimine (PEI), and the anionic center, DNA. In one report, Ghourchian et al. designed an electrochemical assay for the BMP3 gene, a colorectal cancer marker, using PEI–AgNPs as non-specific electroactive labels [58]. A sandwich-type format was employed, with high-affinity capture and reporter probes designed to selectively hybridize with methylated DNA. The resulting complexes produced enhanced current responses, while unmethylated DNA yielded diminished signals. Despite background signal from PEI–AgNPs, thermal optimization steps ensured high specificity, and quantification was achieved in the range of 1 fM to 100 nM.

Zou et al. described an innovative approach for real-time monitoring of methylation levels based on the electron transfer capacity of 6-ferrocenylhexanethiol-modified gold nanoparticles [32]. The strategy involved methylation of DNA by DNMT3a/SAM, followed by bromination with LiBr/NaIO_4_. Bromine’s electron-withdrawing properties attenuated electron density at the CG base pair, effectively suppressing long-range electron transfer. The system demonstrated a sensitivity enhancement from 0.0744 μM to 0.0372 μM upon bromination and was supported by theoretical modeling, highlighting the mechanistic depth of this electrochemical detection approach.

These alternative platforms illustrate the versatility and adaptability of electrochemical methods, showcasing not only high sensitivity and specificity but also novel transduction principles, including adsorption dynamics, strand displacement, magnetic modulation, and redox-tunable surface chemistry. Importantly, many of these strategies have been validated in clinically relevant matrices, reinforcing the growing role of electrochemical biosensing as a translational tool for epigenetic biomarker detection.

## 3. Signal Amplification Strategies for Sensitive DNA Methylation Analysis

**Figure 6:**
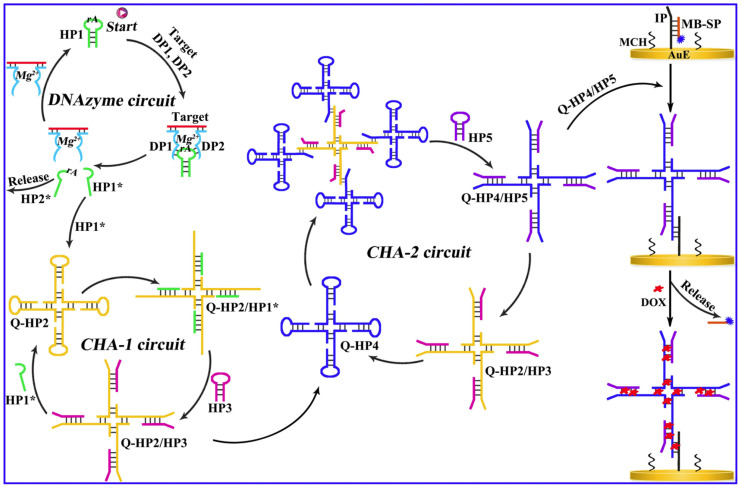
Multistep DNA amplification circuit for methylation detection using DNAzyme-triggered CHA cascades. Reprinted with permission from [59].

The ultrasensitive detection of DNA methylation remains a formidable challenge in clinical diagnostics, primarily due to its extremely low abundance in physiological fluids—typically in the nanogram-per-milliliter range [59]. To address this, a variety of signal amplification strategies have been developed, including polymerase chain reaction (PCR)**,** hybridization chain reaction (HCR)**,** rolling circle amplification (RCA)**,** and catalytic hairpin assembly (CHA)**.** These techniques have proven instrumental in enhancing the sensitivity of electrochemical biosensors [27], particularly when used in tandem with selective DNA recognition interfaces. Among these, CHA amplification has attracted significant interest. It employs hairpin probes with partially complementary sequences that, upon interaction with a specific target, undergo spontaneous conformational changes to form stable duplexes. This reaction is thermodynamically favorable by over two orders of magnitude, facilitating efficient signal generation. The detection performances can be greatly enhanced with the multiple amplification units, where the product of the first cycles initiates the second cycle, and this methodology was utilized by Wang et al. for the aM level determination of DNA methylation ratiometrically employing the redox probes doxorubicin (DOX) and methylene blue (MB) [59]. This procedure involves an upstream DNAzyme and two downstream CHA units. The two segments of the DNAzyme were inserted by the two probe units called DP1 and DP2, which are hybridized with the target methylated DNA. This interaction activated a Mg^2+^-dependent DNAzyme, which cleaved a helper hairpin (HP1) to initiate two successive CHA cascades involving HP2/HP3 and HP4/HP5, respectively. These cascades generated Q-HP4/HP5 complexes, which amplified the signal as a function of the methylated target concentration. The readout relied on the ratiometric electrochemical signals of doxorubicin (DOX) and methylene blue (MB), with the ratio i_DOX_/i_MB_ increasing proportionally with DNA methylation levels. The sensor exhibited a wide linear range (10 aM to 20 pM) and demonstrated high selectivity and clinical relevance, as validated in human serum samples (Figure 6). The high selectivity of the sensor was shown even in the presence of potential interferents and in application studies in human serum samples.

In a complementary strategy, rolling circle amplification (RCA) was employed by Zheng et al. to enable detection across a range of 10^−15^ to 10^−8^ M [60]. RCA is a simple isothermal process that generates long single-stranded DNA concatemers from a circular template. In this case, a linear padlock probe, flanked by target-complementary sequences at both ends, hybridized with methylated DNA to form a circular structure, triggering RCA with a DNA tetrahedron probe. Unmethylated DNA, by contrast, failed to initiate hybridization, allowing for clear differentiation.

The electrode is initially modified with a DNA tetrahedron probe, followed by the RCA reaction with circular padlock probe to generate the G-quadruplex, and finally conjugation with hemin to mimic the HRP-mimic enzyme. The sensing was attributed to the G-quadruplex-hemin DNAzyme catalyzed reduction of H_2_O_2_ via the MB mediators. The resulting RCA product formed G-quadruplex structures, which were complexed with hemin to produce HRP-mimicking DNAzymes. These catalyzed the reduction of H_2_O_2_ via MB mediators, with the current monitored by differential pulse voltammetry (DPV). In the presence of methylated DNA, a threefold signal enhancement was observed relative to unmethylated samples, achieving an LOD of 1 × 10^−16^ M. The system showed excellent reproducibility, selectivity, and stability, reinforcing its value for early diagnosis of epigenetically regulated diseases.

Given the close structural similarity between 5-methylcytosine (5-mC) and 5-hydroxymethylcytosine (5-hmC), their discrimination poses an additional analytical challenge—particularly since standard bisulfite conversion cannot differentiate them [61]. However, 5-hmC is selectively modified by T4 β-glucosyltransferase (T4 β-GT), which transfers a glucose moiety to 5-hmC, leaving 5-mC unaltered. This biochemical distinction has been leveraged in conjunction with restriction endonuclease digestion and HCR signal amplification for enhanced specificity. In this strategy, a gold electrode was modified with dsDNA strands containing both 5-mC and 5-hmC. Electrochemical responses were monitored using the [Ru(NH_3_)_6_]^3+^ redox probe, which binds to DNA via electrostatic interactions. *MspJ*I endonuclease digested both modified bases, initially reducing signal strength. However, the enzymatic conversion of 5-hmC to β-glu-5-hmC by T4 β-GT prevented *MspJI* digestion. In the presence of helper DNAs, this allowed long-chain double helix formation via HCR, accommodating more redox species and significantly amplifying the current response. This efficient coupling of enzyme specificity, DNA structural transformation, and electrochemical transduction enabled not only accurate discrimination between 5-hmC and 5-mC but also femtomolar-level sensitivity, underscoring the strength of integrating bioorthogonal amplification mechanisms with electrochemical readouts.

## 4. Conclusions and Future Perspectives

The detection of DNA methylation has emerged as a powerful strategy for understanding epigenetic mechanisms and developing non-invasive diagnostics for a range of diseases, particularly cancer. This review has highlighted recent advances in electrochemical biosensing platforms for methylation analysis, with an emphasis on bisulfite-free methodologies, direct detection strategies, and signal amplification techniques. These technologies offer several intrinsic advantages, including high sensitivity and specificity, miniaturization potential, low operational cost, and compatibility with complex biological matrices.

Electrochemical detection schemes ranging from direct oxidation of 5-mC, restriction endonuclease-assisted cleavage, and immunoaffinity-based approaches to sophisticated amplification circuits have demonstrated exceptional analytical performance, with detection limits extending to attomolar levels. The incorporation of nanomaterials, magnetic carriers, and bioengineered polymers has further enhanced signal transduction, stability, and target capture efficiency. Importantly, many of these platforms have been successfully validated in clinical samples, underscoring their translational potential for point-of-care diagnostics.

Despite these promising developments, several challenges remain: first, the structural similarity between 5-mC and 5-hmC, the presence of other cytosine modifications, and complicated, unambiguous detection. Innovative biochemical strategies such as selective enzymatic tagging (e.g., T4 β-GT) or redox differentiation will be essential in achieving single-base resolution. Second, the stability and reproducibility of surface modifications, particularly in multiplexed and portable systems, require further standardization for routine use in clinical settings. Third, while many systems demonstrate outstanding performance under laboratory conditions, real-time, high-throughput assays with minimal sample processing remain an unmet need. Looking forward, future research efforts are expected to focus on mainly on the integration with microfluidic devices to create automated, lab-on-chip platforms; coupling with machine learning for advanced data interpretation and classification of methylation signatures; development of universal probe libraries for simultaneous analysis of multiple epigenetic marks; longitudinal monitoring of methylation dynamics in circulating cfDNA for early disease detection and treatment monitoring.

In conclusion, electrochemical biosensors represent a rapidly evolving and versatile technology with transformative implications in epigenetic diagnostics. With continued innovation in biointerface engineering, transducer design, and amplification strategies, these platforms are well-positioned to bridge the gap between benchside discoveries and bedside applications, paving the way for personalized, real-time, and minimally invasive epigenetic screening tools.
